# Th17-Related Cytokines in Systemic Lupus Erythematosus Patients with Dilated Cardiomyopathies: A Possible Linkage to Parvovirus B19 Infection

**DOI:** 10.1371/journal.pone.0113889

**Published:** 2014-12-02

**Authors:** Der-Yuan Chen, Yi-Ming Chen, Bor-Show Tzang, Joung-Liang Lan, Tsai-Ching Hsu

**Affiliations:** 1 Division of Allergy, Immunology and Rheumatology, Department of Medical Education, Taichung Veterans General Hospital, Taichung City, Taiwan; 2 Faculty of Medicine, National Yang Ming University, Taipei, Taiwan; 3 Institute of Biomedical Science and Rong Hsing Research Center for Translational Medicine, National Chung Hsing University, Taichung, Taiwan; 4 Institute of Microbiology and Immunology, Chung Shan Medical University, Taichung, Taiwan; 5 Institute of Biochemistry and Biotechnology, Chung Shan Medical University, Taichung, Taiwan; 6 Department of Biochemistry, School of Medicine, Chung Shan Medical University, Taichung, Taiwan; 7 Immunology Research Center, Chung Shan Medical University, Taichung, Taiwan; 8 Clinical Laboratory, Chung Shan Medical University Hospital, Taichung, Taiwan; 9 Division of Immunology and Rheumatology, China Medical University Hospital, Taichung, Taiwan; 10 College of Medicine, China Medical University, Taichung, Taiwan; University of Michigan Medical School, United States of America

## Abstract

Dilated cardiomyopathies (DCM) are a major cause of mortality in patients with systemic lupus erythematosus (SLE). Immune responses induced by human parvovirus B19 (B19) are considered an important pathogenic mechanism in myocarditis or DCM. However, little is known about Th17-related cytokines in SLE patients with DCM about the linkage with B19 infection. IgM and IgG against B19 viral protein, and serum levels of Th17-related cytokines were determined using ELISA in eight SLE patients with DCM and six patients with valvular heart disease (VHD). Humoral responses of anti-B19-VP1u and anti-B19-NS1 antibody were assessed using Western blot and B19 DNA was detected by nested Polymerase Chain Reaction (PCR). Levels of interleukin (IL)-17, IL-6, IL-1β, and tumor necrosis factor (TNF)-α were significantly higher in SLE patients with DCM (mean ± SEM, 390.99±125.48 pg/ml, 370.24±114.09 pg/ml, 36.01±16.90 pg/ml, and 183.84±82.94 pg/ml, respectively) compared to healthy controls (51.32±3.04 pg/ml, *p*<0.001; 36.88±6.64 pg/ml, *p*<0.001; 5.39±0.62 pg/ml, *p*<0.005; and 82.13±2.42 pg/ml, *p*<0.005, respectively). Levels of IL-17 and IL-6 were higher in SLE patients with DCM versus those with VHD (both *p*<0.01). Five (62.5%) of DCM patients had detectable anti-B19-NS1 IgG and four (50.0%) of them had anti-B19-VP1u IgG, whereas only one (16.7%) of VHD patients had detectable anti-B19-NS1 IgG and anti-B19-VP1u IgG. Serum levels of IL-17, IL-6 and IL-1β were markedly higher in SLE patients with anti-B19-VP1u IgG and anti-B19-NS1 IgG compared to those without anti-B19-VP1u IgG or anti-B19-NS1 IgG, respectively. These suggest a potential association of B19 with DCM and Th17-related cytokines implicated in the pathogenesis of DCM in SLE patients.

## Introduction

Dilated cardiomyopathy (DCM) is one of the most serious forms of organ involvement in systemic lupus erythematosus (SLE) (Doria et al., 2005; [1]–[2]. The most striking symptom of DCM is that of left ventricular (LV) systolic dysfunction and the condition is associated with poor outcome [1]–[2]. Viral infection of the myocardium is now considered the most prevalent cause of DCM resulting from acute myocarditis [3]–[4]. Virus-related DCM is a triphasic disease involving an initial virus infection of myocardium, followed by autoimmune response and finally cardiac injury with dysfunction [5]. In addition to common cardiotropic viruses, such as enteroviruses and adenoviruses [3], [6], human parvovirus B19 (B19) has recently emerged as another common pathogen in these patients with cardiomyopathies, and could be detected in 30–67% of investigated endomyocardial biopsy samples from these patients [4], [7]–[10]. Inflammatory processes induced by viral infection are considered an important pathogenic mechanism in acute myocarditis or DCM, and tumor necrosis factor (TNF)-α and interleukin (IL)-1β may be the dominant inflammatory cytokines expressed in viral myocarditis [11]–[13].

B19 comprises a small non-enveloped particle enclosing a single-stranded linear 5.6-kb DNA genome [14]–[15]. The icosahedral capsid of B19 consists of two structural proteins, VP1 (83 kDa) and VP2 (58 kDa), which are identical except for the 227 amino acids at amino-terminal end of VP1-protein, the so-called VP1-unique region (VP1u) [16]–[17]. B19-VP1u has been demonstrated to have phospholipase A2 (PLA2) activity, which is essential for its cytotoxicity and infectivity [18]–[20]. PLA2 has been associated with macrophage activation and significantly increases IL-6 and IL-1β mRNA expression [21]. We recently demonstrated that B19-VP1u could induce human vascular endothelial cells to produce TNF-α [22], which is known to play an important role in DCM [23]. B19 non-structural (NS1) protein also transactivates the transcription of TNF-α and up-regulates IL-6 transcription [24]. These observations suggest that B19 infection may play a role in cytokine modulation that is related to the pathogenesis of DCM.

T helper type 17 (Th17) cells, a novel distinct subset of Th cell, can secrete IL-17, IL-6, and TNF-α [25]–[27]. IL-17 is a pleiotropic cytokine, which not only plays a role in tissue inflammation [25], but also enhances viral replication [28]–[29]. Elevated levels of serum IL-17 and cardiac IL-17 mRNA, accompanied by the progressive cardiac dysfunction have been observed in coxsackievirus B3-induced acute viral myocarditis [30]. The cardiac pathologic changes were reversed after which the cardiac inflammatory cytokines IL-17, IL-1β, and TNF-α decreased following neutralization of IL-17 [29]–[30]. These observations suggest that Th17-related cytokines are important in the pathogenesis of DCM. However, little is known about Th17-related cytokines in SLE patients complicated with DCM and the possible linkage to B19 infection.

The current investigation examined serum levels of Th17-related cytokines in SLE patients with DCM. This study enrolled SLE patients with valvular heart disease (VHD) as disease control because no documented association of B19 infection with this complication. Additionally, this study examined the association of Th17-related cytokines with the occurrence of DCM and the presence of B19 infection in such patients.

## Materials and Methods

### Patients and Sera

Fourteen SLE patients [31] with cardiac dysfunction (all females mean age 35.6±8.6 years) were enrolled from Taichung Veterans General Hospital. The enrolled subjects were classified into two groups: SLE with DCM [32] and SLE with VHD [1], based on clinical features and echocardiographic findings [33]. The clinical diagnosis of DCM was made in patients who presented with global LV dysfunction (ejection fraction <45%) and/or dilated LV in association with symptoms of heart failure [7], [32]. All patients with coronary artery disease or other possible causes of cardiac dysfunction were excluded. Ten sex- and age-matched healthy volunteers (10 females, mean age 33.2±4.5 years), who had no rheumatic disease, were used as normal controls. The blood samples were centrifuged at 1250 rpm for 10 min and the sera were collected and stored at −70°C until examination. This study was approved by the Institutional Review Board of Taichung Veterans General Hospital (approval number: 940123/528), and each participant's written consent was obtained according to the Declaration of Helsinki.

### Detection of IgM and IgG against B19-VP in serum from SLE patients

The IgM and IgG against B19-VP were analyzed by enzyme-linked immunosorbent assay (ELISA) according to the manufacturer's instructions (IBL-America, MN, USA). Standard, control, and diluted samples were included in each plate. The absorbance (O.D.) was read at the wavelength of 450/620 nm after adding 100 µl of stop solution. According to the product information provided by manufacture, the sensitivity of anti-VP ELISA is 98 to 100%. Negative values range from 0–11 U (B19 IgM and B19 IgG). Positive results are greater than 12 U (B19 IgM and B19 IgG).

### Determination of IgG against B19-VP1u and B19-NS1 using Immunoblotting

Sodium dodecyl sulfate-polyacrylamide gel electrophoresis (SDS-PAGE), using a 12.5% acrylamide slab gel with 5% acrylamide stacking gel, was performed according to the method of Laemmli [34]. Samples were reduced for 5 min in boiling water with 0.0625 M Tris-HCl buffer, pH 6.8, containing 2.3% SDS, 5% 2-mercaptoethanol, and 10% glycerol. Samples and pre-stained protein marker (FERMENTAS Life Science, CA, USA) were applied to the gel and electrophoresis was performed at 100–150 V for 1.5 hr. The samples were electrophoretically transferred to nitrocellulose, according to the method of Towbin [35]. Human or rabbit antiserum against B19-VP1u or B19-NS1 was diluted with 5% nonfat dry milk in PBS, reacted with the nitrocellulose strips and then incubated for 1.5 hr at room temperature. The strips were washed twice with PBS-Tween for 1 hr and secondary antibody consisting of alkaline phosphatase conjugated goat anti-human or rabbit IgG antibodies was added. The substrate NBT/BCIP (nitroblue tetrazolium/5-bromo-4-chloro-3 indolyl phosphate) was used to detect antigen-antibody complexes.

### DNA extraction, PCR amplification, and detection of B19 genomes

DNA was extracted from serum by using a QIA Amp blood kit (QIAagen, Hilden, Germany) as directed by the manufacturer. In the first round of PCR amplification, 0.2 mM of nucleotide primers corresponding to nucleotide (nt) 2381–2400 (B19SI) and nt 2781-2800 (B19ASI) (5′-CCTTTTCTGTGCTAACCTGC-3′ and 5′-CCCAGGCTTGTGTAAGTCTT-3′, respectively) were used. Two µl of each sample were used in a 50 µl reaction containing 5 µl of 10× buffer (500 mM Tris-HCl pH 8.7, 50 mM NH_4_Cl, 20 mM MgCl_2_, 400 mM KCl, 1% Triton X-100), 4 µl of 25 mM dNTP, 2.5 U of Taq DNA polymerase (Takara, Tokyo, Japan) and 36 µl sterilized water. After an initial denaturation step of 5 min at 94°C, thirty-five cycles were performed at 94°C for 45 seconds, 54°C for 45 seconds, and 72°C for 1 min. After the first round amplification, 2 µl of the first PCR product were added to the second round PCR mixture containing 2 mM of each oligonucleotide primer corresponding to nucleotide 2429–2448 (B19SII) and nucleotide 2730–2751 (B19ASII) (5′-AAAGCTTTGTAGATTATGAG-3′and 5′-GGTTCTGCATGACTGCTATG G-3′). Then thirty-five cycles of amplification were performed using the described cycling parameters. Subsequently, the nested PCR products of size 322 bp nucleotides and GelPilot 100 bp Plus Ladder (Qiagen, Chatsworth, CA, USA) were electrophoresed on a 1% agarose gel in TAE buffer and visualized under ultraviolet (UV) light after staining with ethidium bromide. B19 positive and negative reference controls were also included in each PCR reaction. The nested PCR was used as it eliminates non-specific background and thus gives a clearer final product.

### Determination of serum levels of Th17-related cytokines

Serum levels of IL-1β, IL-6, IL-17, and TNF-α were determined using ELISA according to the manufacturer's instructions (eBiosciences, San Diego, USA).

### Statistical analyses

Data were analyzed using SPSS 10.0 for windows (Chicago, IL, USA). The nonparametric Kruskal-Wallis test and Mann-Whitney U test were used for between-group comparison of serum levels of IL-17, IL-6, IL-1β, and TNF-α. P value <0.05 was considered to be statistically significant.

## Results

### Demographic data, clinical characteristics, and laboratory findings in SLE patients

 As illustrated in Table 1, all SLE patients with cardiac dysfunction were female. The most common cardiac symptom was dyspnea in SLE patients with DCM (100%) and SLE patients with VHD (50%). No significant difference in age at onset of disease, disease duration, lupus manifestations, laboratory findings, disease activity, daily dose of corticosteroid, or the proportion of used immunosuppressive agents were observed between the SLE patients with DCM and SLE patients with VHD.

**Table 1:** Demographic data and clinical characteristics of SLE patients with dilated cardiomyopathies (DCM) and valvular heart diseases (VHD).^a^

Characteristics	DCM	VHD
	(n = 8)	(n = 6)
Age at onset of cardiac symptoms (years)	36.9±10.0	33.8±6.6
Females	8 (100%)	6 (100%)
Duration of diseases (years)	6.3±1.5	7.0±1.8
Malar rash	8 (100%)	5 (83.3%)
Arthritis	6 (75.0%)	4 (66.7%)
Nephritis	2 (25.0%)	3 (50.0%)
CNS involvement	1 (12.5%)	2 (33.3%)
Raynaud's phenomenon	6 (75.0%)	4 (66.7%)
Pulmonary hypertension	4 (50.0%)	1 (20.0%)
Leukopenia (<4000/mm^2^)	3 (37.5%)	3 (50.0%)
Anemia (<11.3 mg/dl)	6 (75.0%)	4 (66.7%)
Thrombocytopenia(<1×10^5^/mm^2^)	2 (25.0%)	2 (33.3%)
Cardiac symptoms		
Dyspnea	8 (100%)	3 (50.0%)
Angina	3 (37.5%)	2 (33.3%)
Lower legs edema	3 (37.5%)	1 (16.7%)
Serum C3 levels (mg/dl)	46.5±16.7	68.0±9.8
Serum C4 levels (mg/dl)	8.1±4.1	11.7±3.5
Anti-ds DNA (U/ml)	167.4±65.2	150.0±112.4
SLEDAI	21.1±6.5	17.3±4.3
Daily dose of corticosteroid (mg)	22.5±6.5	20.0±5.5
Use of oral immunosuppressive agents b	8 (100%)	5 (83.3%)

SLE: systemic lupus erythematosus; Nephritis was defined as persistent proteinuria (>0.5 g/24 hours) or pathological confirmation of renal biopsy specimens showing lupus nephritis; C3: complement 3; C4: complement 4; Anti-dsDNA: anti-double strand DNA antibody; SLEDAI: SLE disease activity index.

a Data are presented as mean ±SD or number (percentage).

b Include hydroxychloroquine, azathioprine, cyclophosphamide, mycophenolate mofetil or ciclosporine.

### Serum levels of Th17-related cytokines in SLE patients with DCM and VHD

**Figure 1:**
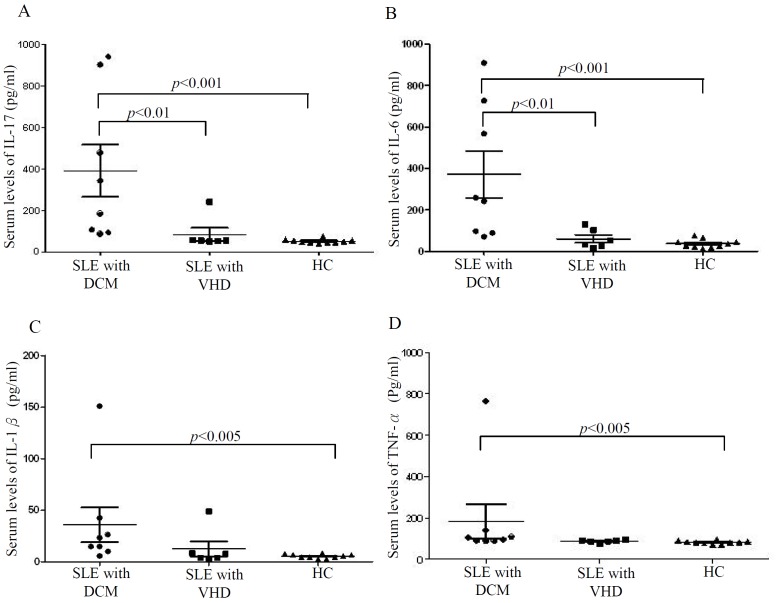
Serum levels of (A) IL-17, (B) IL-6, (C) IL-1β, and (D) TNF-α in eight patients with systemic lupus erythematosus (SLE) complicated with dilated cardiomyopathy (DCM) and six SLE patients with valvular heart disease (VHD). The data are presented as dot-plot diagrams with mean ± SEM.

As illustrated in Figure 1, serum levels of IL-17, IL-6, IL-1β, and TNF-α were significantly higher in SLE patients with DCM (mean ± SEM, 390.99±125.48 pg/ml, 370.24±114.09 pg/ml, 36.01±16.90 pg/ml, and 183.84±82.94 pg/ml, respectively) compared to healthy controls (51.32±3.04 pg/ml, p<0.001; 36.88±6.64 pg/ml, p<0.001; 5.39±0.62 pg/ml, p<0.005; and 82.13±2.42 pg/ml, p<0.005, respectively). Serum levels of IL-17 and IL-6 were also markedly higher in SLE patients with DCM than in those with VHD (84.39±31.43 pg/ml and 59.73±18.72 pg/ml, respectively, both p<0.01). No significant differences in serum levels of IL-17, IL-6, IL-1β, and TNF-α existed between SLE patients with VHD and healthy controls.

### The association of B19 infection with DCM in SLE patients

Based on the advances in diagnosis of B19 infection [15], one (12.5%) of the eight SLE patients with DCM had detectable B19 DNA, indicating persistent infection. Additionally, B19-NS1 antibodies have been observed to be more prevalent in patients with persistent B19 viremia or delayed control of viremia following acute infection, indicating a persisting B19 infection [36]. Five (62.5%) of the eight SLE patients with DCM had detectable anti-B19-NS1 IgG and four (50.0%) had anti-B19-VP1u IgG, whereas only one (16.7%) of the six SLE patients with VHD had detectable anti-B19-NS1 IgG and anti-B19-VP1u IgG.

### Levels of Th17-related cytokines in SLE patients with and without B19 infection

**Figure 2:**
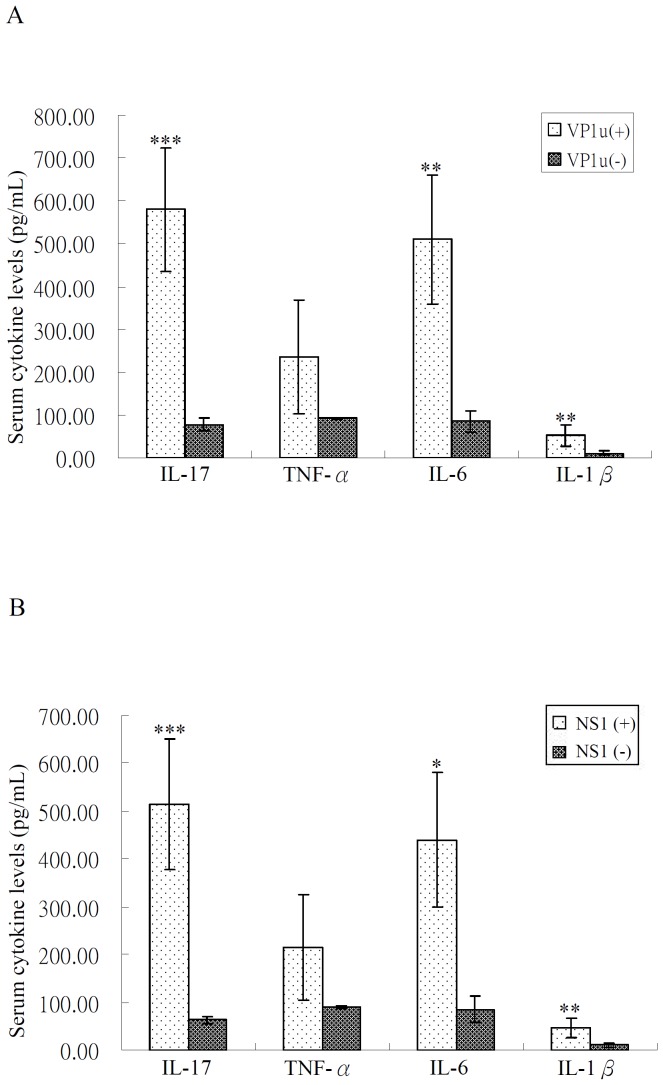
The difference in the levels of IL-17, IL-6, IL-1β and TNF-α (A) between SLE patients with anti-B19-VP1u IgG [VP1u (+)] and without anti-B19-VP1u IgG [VP1u (−)], and (B) between SLE patients with anti-B19-NS1 IgG [NS1 (+)] and without anti-B19-NS1 IgG [NS1(−)]. Data are presented as mean ± SEM. *p<0.05, **p<0.01, ***p<0.005, versus SLE patients without anti-B19-VP1u IgG or anti-B19-NS1 IgG.

As illustrated in Figure 2, serum levels of IL-17, IL-6, and IL-1β were significantly higher in SLE patients with anti-B19-VP1u IgG (mean ± SEM, 580.70±144.36 pg/ml, 509.66±149.64 pg/ml, and 52.80±25.16 pg/ml, respectively) compared to those without anti-B19-VP1u IgG (77.98±15.89 pg/ml, p<0.005; 85.77±24.89 pg/ml, p<0.01; and 11.12±4.12 pg/ml, p<0.01, respectively). Serum levels of IL-17, IL-6, and IL-1β were also significantly higher in SLE patients with anti-B19-NS1 IgG (514.19±135.34 pg/ml, 439.58±140.85 pg/ml, and 46.44±21.51 pg/ml, respectively) than in those without anti-B19-NS1 IgG (63.17±6.66 pg/ml, p<0.005; 85.34±28.22 pg/ml, p<0.05; and 10.69±4.65 pg/ml, p<0.01, respectively). Serum levels of TNF-α did not differ significantly between SLE patients with and without B19 infection.

## Discussion

This study is the first to investigate serum levels of Th17-related cytokines in SLE patients with DCM and examined the possible linkage to B19 Infection. The study results show significantly higher levels of serum Th17-related cytokines, including IL-17, IL-6, IL-1β and TNF-α in SLE patients with DCM compared to healthy controls, indicating the existence of immune responses in DCM patients. The study data support the findings of a recent study showing that Th17 cells facilitate the immune response in patients with viral myocarditis [28]. Significantly elevated IL-17 levels the SLE patients with DCM were consistent with the findings of a recent study demonstrating that IL-17 is essential for postmyocarditis cardiac remodeling and the progression to DCM [37]. The results of this study were also in agreement with the results of Daniels et al. showing that Th17 cells implicated in the myocardial inflammation in experimental autoimmune myocarditis, mimicking viral myocarditis [38]. The study data also supported the findings of animal studies demonstrating the therapeutic benefits of blockade of IL-17 via monoclonal antibodies [29]–[30]. These observations strongly suggest a potential role for Th17-related cytokines in the pathogenesis of SLE with DCM. However, it has recently been demonstrated using flow cytometry analysis that circulating Th17 cells significantly increased in patients with acute vial myocarditis, while circulating Th2 cells significantly increased in patients with DCM compared with those of healthy controls [28]. This disparity in the profile of circulating cytokine may be due to differences in population characteristics and detection method.

IL-1β and IL-6 have been reported to induce the expression of retinoic acid receptor-related orphan receptor (RORγt) and stimulate the differentiation of Th17 cells from naïve T cells [39]. Elevated levels of serum IL-6 have been observed in myocarditis patients compared to healthy controls, and have been related to the degree of cardiac dysfunction and overall poor outcome [40]. Additionally, IL-1β, a potent profibrotic cytokine, is critical for the development of chronic inflammatory heart disease including DCM [41]. Previous investigations showed that the neutralization of IL-1β with an IL-1β receptor antagonist could decrease myocardial injury in a murine model of viral myocarditis [42]. The investigation showed that levels of serum IL-6 and IL-1β were significantly elevated in SLE patients with DCM, suggesting Th17-related cytokines are important in the immune response of this disease.

A recent study showed that B19 was the most frequent pathogen in the myocardium of adults with DCM [4], [7], [32]. Pankuweit et al. also reported that B19 was the most common detected virus in 3345 patients with cardiomyopathies, with positivity ranging from 17.6–33.3% in patients with non-inflammatory cardiomyopathies, inflammatory cardiomyopathies and DCM [8]. Moreover, there was a positive correlation between detection of B19 and occurrence of cardiac dysfunction in patients with inflammatory cardiomyopathies [8]. In the present study, we found a higher positive rate for anti-B19-NS1 IgG and anti-B19-VP1u IgG in SLE patients with DCM compared to those with valvular lesion, suggesting possible involvement of B19 infection in the pathogenesis of DCM. Both the findings of our study and previous reports suggested that B19 is a potential important aetiological factor in SLE patients with DCM. In SLE patients with positivity for anti-B19-VP1u or anti-B19-NS1 antibodies and cardiac dysfunction, the presence of viral DCM should be considered. In our study, the absence of detectable B19 genome or anti-B19-VP IgM in serum from SLE patients with DCM supports the hypothesis that myocardial inflammatory/immune response occurs in a few DCM patients despite virus elimination [43]. Whether B19 plays a pathogenic role or serves as an innocent bystander remains debatable.

Significantly higher levels of Th17-related cytokines were observed in the SLE patients with positive B19-VP1u antibody compared to those without B19-VP1u antibody, suggesting VP1u may influence the modulation of inflammatory reactions through its PLA2 activity [20]. Among the SLE patients with DCM, considerably raised levels of Th17-related cytokines were also observed in those with B19-NS1 antibody versus those without B19-NS1 antibody. Our results were in agreement with the findings of previous studies demonstrating that B19 NS1 protein is a transactivator of IL-6 expression [44]–[45], indicating that NS1 is important in the B19-related diseases.

The limitations of this study are its retrospective design and the relatively small number of patients enrolled. Additionally, this study did not investigate the presence of anti-heart autoantibodies. However, it remains uncertain whether these autoantibodies cause cardiomyopathies or appear as the result of myocardial injury. Despite these limitations, the study findings may help understand the potential roles of Th17-related cytokines in the immune response and the possible linkage with B19 infection in SLE-related DCM. The aetiopathogenesis of DCM in SLE patients is complicated, and future research should address whether B19 is a direct pathogen or bystander, or is just the adjuvant for autoimmunity in viral myocarditis [46].

## Conclusions

The study results suggest that Th17-related cytokines may be involved in the pathogenesis of DCM in SLE patients. The overproduction of Th17-related cytokines may be an important therapeutic target for SLE-related DCM. A potential association of B19 with DCM is supported by the higher positive rate of B19-specific antibodies in SLE patients with DCM versus those with VHD. Awareness of probability of B19-related DCM will enable clinicians to test for B19 infection and avoid the use of aggressive immunosuppressant in B19-infected patients with DCM. Additionally, B19-VP1u may be a future target for neutralizing antibodies or DNA vaccine.

## References

[1] Doria A, Iaccarino L, Sarzi-Puttini P, Atzeni F, Turriel M, et al (2005) Cardiac involvement in systemic lupus erythematosus. Lupus 14:683–686. 16218467 10.1191/0961203305lu2200oa

[2] Manger K, Manger B, Repp R, Geisselbrecht M, Geiger A, et al (2002) Definition of risk factors for death, end stage renal disease, and thromboembolic events in a monocentric cohort of 338 patients with systemic lupus erythematosus. Ann Rheum Dis 61:1065–1070. 12429536 10.1136/ard.61.12.1065 PMC1753955

[3] Pauschinger M, Bowles NE, Fuentes-Garcia FJ, Pham V, Kühl U, et al (1999) Detection of adenoviral genome in the myocardium of adult patients with idiopathic left ventricular dysfunction. Circulation 99:1348–1354. 10077520 10.1161/01.cir.99.10.1348

[4] Andréoletti L, Lévêque N, Boulagnon C, Brasselet C, Fornes P (2009) Viral causes of human myocarditis. Arch Cardiovasc Dis 102:559–568. 19664576 10.1016/j.acvd.2009.04.010

[5] Dennert R, Crijns HJ, Heymans S (2008) Acute viral myocarditis. Eur Heart J 29:2073–2082. 18617482 10.1093/eurheartj/ehn296 PMC2519249

[6] Feldman AM, McNamara D (2000) Myocarditis. N Engl J Med 343:1388–1398. 11070105 10.1056/NEJM200011093431908

[7] Escher F, Modrow S, Sabi T, Kühl U, Lassner D, et al (2008) Parvovirus B19 profiles in patients presenting with acute myocarditis and chronic dilated cardiomyopathy. Med Sci Monit 14:CR589–597. 19043365

[8] Pankuweit S, Moll R, Baandrup U, Portig I, Hufnagel G, et al (2003) Prevalence of the parvovirus B19 genome in endomyocardial biopsy specimens. Hum Pathol 34:497–503. 12792925 10.1016/s0046-8177(03)00078-9

[9] Kühl U, Lassner D, Pauschinger M, Gross UM, Seeberg B, et al (2008) Prevalence of erythrovirus genotypes in the myocardium of patients with dilated cardiomyopathy. J Med Virol 80:1243–1251. 18461615 10.1002/jmv.21187

[10] Tschöpe C, Bock CT, Kasner M, Noutsias M, Westermann D, et al (2005) High prevalence of cardiac parvovirus B19 infection in patients with isolated left ventricular diastolic dysfunction. Circulation 111:879–886. 15710767 10.1161/01.CIR.0000155615.68924.B3

[11] Yamada T, Matsumori A, Sasayama S (1994) Therapeutic effect of anti-tumor necrosis factor-alpha antibody on the murine model of viral myocarditis induced by encephalomyocarditis virus. Circulation 89:846–851. 8313574 10.1161/01.cir.89.2.846

[12] Huber SA, Polgar J, Schultheiss P, Schwimmbeck P (1994) Augmentation of pathogenesis of coxsackievirus B3 infections in mice by exogenous administration of interleukin-1 and interleukin-2. J Virol 68:195–206. 8254729 10.1128/jvi.68.1.195-206.1994 PMC236278

[13] Tzang BS, Lin TM, Tsai CC, Hsu JD, Yang LC, et al (2011) Increased cardiac injury in NZB/W F1 mice received antibody against human parvovirus B19 VP1 unique region protein. Mol Immuno 48:1518–1524. 10.1016/j.molimm.2011.04.013 21555155

[14] Corcoran A, Doyle S (2004) Advances in the biology, diagnosis and host-pathogen interactions of parvovirus B19. J Med Microbiol 53:459–475. 15150324 10.1099/jmm.0.05485-0

[15] Young NS, Brown KE (2004) Parvovirus B19. N Engl J Med 350:586–597. 14762186 10.1056/NEJMra030840

[16] Anderson S, Momoeda M, Kawase M, Kajigaya S, Young NS (1995) Peptides derived from the unique region of B19 parvovirus minor capsid protein elicit neutralizing antibodies in rabbits. Virology 206:626–632. 7530397 10.1016/s0042-6822(95)80079-4

[17] Musiani M, Manaresi E, Gallinella G, Venturoli S, Zuffi E, et al (2000) Immunoreactivity against linear epitopes of parvovirus B19 structural proteins. Immunodominance of the amino-terminal half of the unique region of VP1. J Med Virol 60:347–352. 10630969 10.1002/(sici)1096-9071(200003)60:3<347::aid-jmv15>3.0.co;2-t

[18] Zádori Z, Szelei J, Lacoste MC, Li Y, Gariépy S, et al (2001) A viral phospholipase A2 is required for parvovirus infectivity. Dev Cell 1:291–302. 11702787 10.1016/s1534-5807(01)00031-4

[19] Leisi R, Ruprecht N, Kempf C, Ros C (2013) Parvovirus B19 uptake is a highly selective process controlled by VP1u, a novel determinant of viral tropism. J Virol 87:13161–13167. 24067971 10.1128/JVI.02548-13 PMC3838272

[20] Dorsch S, Liebisch G, Kaufmann B, von Landenberg P, Hoffmann JH, et al (2002) The VP1 unique region of parvovirus B19 and its constituent phospholipase A2-like activity. J Virol 76:2014–2018. 11799199 10.1128/JVI.76.4.2014-2018.2002 PMC135890

[21] Tzang BS, Chiu CC, Tsai CC, Lee YJ, Lu IJ, et al (2009) Effects of human parvovirus B19 VP1 unique region protein on macrophage responses. J Biomed Sci 16:13. 19272185 10.1186/1423-0127-16-13 PMC2653524

[22] Tzang BS, Tsai CC, Chiu CC, Shi JY, Hsu TC (2008) Up-regulation of adhesion molecule expression and induction of TNF-α on vascular endothelial cell by antibody against human parvovirus B19 VP1 unique region protein. Clin Chim Acta 395:77–83. 18538665 10.1016/j.cca.2008.05.012

[23] Satoh M, Nakamura M, Saitoh H, Satoh H, Maesawa C, et al (1999) Tumor necrosis factor-alpha-converting enzyme and tumor necrosis factor-alpha in human dilated cardiomyopathy. Circulation 99:3260–3265. 10385500 10.1161/01.cir.99.25.3260

[24] Moffatt S, Tanaka N, Tada K, Nose M, Nakamura M, et al (1996) A cytotoxic nonstructural protein, NS1, of human parvovirus B19 induces activation of interleukin-6 gene expression. J Virol 70:8485–8491. 8970971 10.1128/jvi.70.12.8485-8491.1996 PMC190939

[25] Park H, Li Z, Yang XO, Chang SH, Nurieva R, et al (2005) A distinct lineage of CD4 T cells regulates tissue inflammation by producing interleukin 17. Nat Immunol 6:1133–1141. 16200068 10.1038/ni1261 PMC1618871

[26] Harrington LE, Hatton RD, Mangan PR, Turner H, Murphy TL, et al (2005) Interleukin 17-producing CD4+ effector T cells develop via a lineage distinct from the T helper type 1 and 2 lineages. Nat Immunol 6:1123–1132. 16200070 10.1038/ni1254

[27] Korn T, Oukka M, Kuchroo V, Bettelli E (2007) Th17 cells: effector t cells with inflammatory properties. Semin Immunol 19:362–371. 18035554 10.1016/j.smim.2007.10.007 PMC2839934

[28] Yuan J, Cao AL, Yu M, Lin QW, Yu X, et al (2010) Th17 cells facilitate the humoral immune response in patients with acute viral myocarditis. J Clin Immunol 30:226–234. 20012175 10.1007/s10875-009-9355-z

[29] Yuan J, Yu M, Lin QW, Cao AL, Yu X, et al (2010) Neutralization of IL-17 inhibits the production of anti-ANT autoantibodies in CVB3-induced acute viral myocarditis. Int Immunopharmacol 10:272–276. 19932195 10.1016/j.intimp.2009.11.010

[30] Yuan J, Yu M, Lin QW, Cao AL, Yu X, et al (2010) Th17 cells contribute to viral replication in coxsackievirus B3-induced acute viral myocarditis. J Immunol 185:4004–4010. 20802148 10.4049/jimmunol.1001718

[31] Hochberg MC (1997) Updating the American College of Rheumatology revised criteria for the classification of systemic lupus erythematosus. Arthritis Rheum 40:1725–1729. 10.1002/art.1780400928 9324032

[32] Kühl U, Pauschinger M, Noutsias M, Seeberg B, Bock T, et al (2005) High prevalence of viral genomes and multiple viral infections in the myocardium of adults with "idiopathic" left ventricular dysfunction. Circulation 111:887–893. 15699250 10.1161/01.CIR.0000155616.07901.35

[33] Omdal R, Lunde P, Rasmussen K, Mellgren SI, Husby G (2001) Transesophageal and transthoracic echocardiography and Doppler-examinations in systemic lupus erythematosus. Scand J Rheumatol 30:275–281. 11727842 10.1080/030097401753180354

[34] Laemmli UK (1970) Cleavage of structural proteins during the assembly of the head of bacteriophage T4. Nature 227:680–685. 5432063 10.1038/227680a0

[35] Towbin H, Staehelin T, Gordon J (1979) Electrophoretic transfer of proteins from polyacrylamide gels to nitrocellulose sheet: procedure and some applications. Proc Natl Acad Sci USA 76:4350–4354. 388439 10.1073/pnas.76.9.4350 PMC411572

[36] Kerr JR, Cunniffe VS (2000) Antibodies to parvovirus B19 non-structural protein are associated with chronic but not acute arthritis following B19 infection. Rheumatology (Oxford) 39:903–908. 10952747 10.1093/rheumatology/39.8.903

[37] Baldeviano GC, Barin JG, Talor MV, Srinivasan S, Bedja D, et al (2010) Interleukin-17A is dispensable for myocarditis but essential for the progression to dilated cardiomyopathy. Circ Res 106:1646–1655. 20378858 10.1161/CIRCRESAHA.109.213157

[38] Daniels MD, Hyland KV, Wang K, Engman DM (2008) Recombinant cardiac myosin fragment induces experimental autoimmune myocarditis via activation of Th1 and Th17 immunity. Autoimmunity 41:490–499. 18781477 10.1080/08916930802167902 PMC2702149

[39] Zhou L, Ivanov II, Spolski R, Min R, Shenderov K, et al (2007) IL-6 programs T(H)-17 cell differentiation by promoting sequential engagement of the IL-21 and IL-23 pathways. Nat Immunol 8:967–974. 17581537 10.1038/ni1488

[40] Kanda T, Takahashi T (2004) Interleukin-6 and cardiovascular diseases. Jpn Heart J 45:183–193. 15090695 10.1536/jhj.45.183

[41] Francis SE, Holden H, Holt CM, Duff GW (1998) Interleukin-1 in myocardium and coronary arteries of patients with dilated cardiomyopathy. J Mol Cell Cardiol 30:215–223. 9514998 10.1006/jmcc.1997.0592

[42] Neumann DA, Lane JR, Allen GS, Herskowitz A, Rose NR (1993) Viral myocarditis leading to cardiomyopathy do cytokines contribute to pathogenesisis? Clin Immunol Immunopathol 68:181–190. 8395360 10.1006/clin.1993.1116

[43] Noutsias M, Hohmann C, Pauschinger M, Schwimmbeck PL, Ostermann K, et al (2003) sICAM-1 correlates with myocardial ICAM-1 expression in dilated cardiomyopathy. Int J Cardiol 91:153–161. 14559125 10.1016/s0167-5273(03)00033-0

[44] Mitchell LA (2002) Parvovirus B19 nonstructural (NS1) protein as a transactivator of interleukin-6 synthesis: common pathway in inflammatory sequelae of human parvovirus infections? J Med Virol 67:267–274. 11992589 10.1002/jmv.2217

[45] Hsu TC, Tzang BS, Huang CN, Lee YJ, Liu GY, et al (2006) Increased expression and secretion of interleukin-6 in human parvovirus B19 non-structural protein (NS1) transfected COS-7 epithelial cells. Clinical and Experimental Immunology 144:152–157. 16542377 10.1111/j.1365-2249.2006.03023.x PMC1809635

[46] Fairweather D, Frisancho-Kiss S, Rose NR (2005) Viruses as adjuvants for autoimmunity: evidence from Coxsackievirus-induced myocarditis. Rev Med Virol 15:17–27. 15386590 10.1002/rmv.445

